# T Cell-Mediated Immunity towards Yellow Fever Virus and Useful Animal Models

**DOI:** 10.3390/v9040077

**Published:** 2017-04-11

**Authors:** Alan M. Watson, William B. Klimstra

**Affiliations:** Center for Vaccine Research, Departments of Microbiology and Molecular Genetics, and Immunology, University of Pittsburgh, 3501 Fifth Avenue, Pittsburgh, PA 15261, USA; klimstra@pitt.edu

**Keywords:** yellow fever virus, YFV, flavivirus, 17D, 17DD, 17D-204, T cell, vaccine, animal models, dengue, West Nile, Zika, live attenuated vaccine, chimerivax, vaccine development

## Abstract

The 17D line of yellow fever virus vaccines is among the most effective vaccines ever created. The humoral and cellular immunity elicited by 17D has been well characterized in humans. Neutralizing antibodies have long been known to provide protection against challenge with a wild-type virus. However, a well characterized T cell immune response that is robust, long-lived and polyfunctional is also elicited by 17D. It remains unclear whether this arm of immunity is protective following challenge with a wild-type virus. Here we introduce the 17D line of yellow fever virus vaccines, describe the current state of knowledge regarding the immunity directed towards the vaccines in humans and conclude with a discussion of animal models that are useful for evaluating T cell-mediated immune protection to yellow fever virus.

## 1. Yellow Fever Virus: History, Legacy and Future

### 1.1. Introduction to the Yellow Fever Virus

Yellow fever virus (YFV) simultaneously shares an important historical legacy and a modern-day urgency. Once one of the most feared diseases in the world, this virus terrorized Africa, Europe and the Americas. In the Americas, over 250 years of documented epidemics of YFV killed hundreds of thousands and influenced the outcomes of wars and the stability of economies. YFV is the prototypic member of the genus *Flavivirus*, family *Flaviviridae*, and contains a single-stranded, positive sense RNA genome. In the urban YFV cycle, in which most human cases occur, the virus is transmitted by the bite of an infected *Aedes aegypti* mosquito. A subset of infected people develops a severe hemorrhagic yellow fever (YF) disease, presenting with fever, nausea, vomiting, hepatitis, and jaundice. This severe disease leads to death in 20%–60% of cases (reviewed in [1]).

Originating in Africa, YFV was trafficked to the Americas as a consequence of the slave trade [2]. Eventually, better sanitation led to a precipitous decline in outbreaks of YF. Even as local outbreaks decreased, YFV remained a threat to the United States because of foreign conflicts and foreign economic development. Two prominent examples of this include Cuba during the Spanish-American war where YF killed more American soldiers than battle, and the construction of the Panama Canal which was devastated by ongoing outbreaks of YF. Following the end of the Spanish-American war, YF remained a concern to the United States regarding both the protection of soldiers during foreign conflicts and the possibility of domestic outbreaks. The U.S. Army’s Yellow Fever Commission, led famously by Walter Reed, traveled to Cuba and established that mosquitoes were responsible for transmission [3]. Subsequently, mosquito control efforts were used to reduce the impact of the last major U.S. epidemic in New Orleans in 1905, and bring an end to the outbreaks at the Panama Canal in 1906.

### 1.2. A Brief History of the Yellow Fever Virus Vaccine

In the four decades following the yellow fever commission, an international effort developed to isolate, propagate and create a vaccine against YFV. Integral to this effort was the development of animal models that were required to produce a vaccine. During the fall of 1925 Adrian Stokes led an expedition to study yellow fever in West Africa. In the course of their studies they isolated a virulent virus from a Ghanaian man named Asibi with a mild case of YF [4,5]. The Asibi virus was passaged through rhesus macaques by direct blood/serum transfer and then through infected mosquitoes. Except for two monkeys, the Asibi virus proved lethal causing symptoms that were reportedly similar to human cases of yellow fever. The studies carried out by Stoke’s expedition were ground-breaking on various levels as they were the first to establish experimental animal models of YF and show that serum from convalescent humans could protect experimentally infected animals.

The Asibi virus was transported to the Rockefeller Institute where Max Theiler and colleagues discovered that the virus, which was refractory to growth in small laboratory animals through most routes of injection, would grow in the brains of mice following intracranial injection [6], the first record of mice being used as an animal model. Passage in mouse brains reduced the viscerotropic virulence of the virus in monkeys but enhanced the neurotropic properties, causing lethal disease when injected into the brain [7]. Concerns over neurotropism led Theiler’s group to passage the virus over 200 times in tissue culture medium made with chicken embryos from which the neurologic tissue was removed. They designated one subculture of the Asibi virus, 17D. Although the 17D culture remained virulent when injected into mouse brains, the virus had lost its neurovirulence in monkeys, causing only a ‘moderate febrile reaction’ when injected intracerebrally [8]. Moreover, the virus no longer caused viscertropic disease in monkeys when injected subcutaneously but only a very mild infection.

Simultaneously with the above findings, Theiler published a report showing that when the 17D subculture was inoculated into monkeys, immune serum could be detected within one month of infection. Within seven days of infection, the monkeys were completely protected against challenge with the virulent Asibi virus. At seven days and beyond, no circulating Asibi virus was detected in the blood of vaccinated monkeys. In humans injected with 17D, anti-yellow fever immune serum was detected as early as two days following immunization. The eight test subjects experienced only a slight ‘fever’ (maximum temperature 37.4 °C), a mild headache and a backache that reportedly did not prevent normal daily activities [9]. The 17D subculture of the Asibi virus [8] became the seed strain for the modern day yellow fever virus vaccines, 17DD (passage 195) and 17D-204 (passage 204). Since then, over 500 million people have been administered the 17D-based vaccines (hereafter referred to collectively as 17D). Remarkably, only 32 cases of vaccine failure to protect against a virulent strain of YFV have ever been documented [1]. This is likely due to the high seroconversion rate of human vaccinees, between 80% and 100%, and the 30-year persistence of detectable immunity in some individuals. Max Theiler was awarded the Nobel prize in 1951 [10].

For a more through appreciation of the history of the yellow fever vaccine, the authors suggest reading the excellent discussion by J. Gordon Frierson [11] and the thorough review by Monath, Gershman and Staples [1].

### 1.3. Status of the Yellow Fever Virus and 17D

Today, YFV remains endemic in South American and African countries where monkeys provide sylvatic reservoirs of virus that spur regular outbreaks (jungle yellow fever). Estimates are as high as 200,000 infections with YFV per year and up to 30,000 deaths [12]. In these countries, vaccination against the virus is a primary means of disease control. However, the virus remains of major concern in Sub-Saharan Africa where the prevalence of vaccination is low and millions are at risk for infection. Indeed, in 2016 Angola experienced its biggest outbreak of YFV in 30 years and the virus has spread to neighboring countries and resulted in numerous traveler-associated cases throughout the globe. Most notably, YFV had never been recorded in Asia; however, China experienced its first traveler-associated cases in 2016 [13]. Due to high population densities and sufficient mosquito populations, yellow fever could be particularly devastating in Asia if it were to become established [14]. The reintroduction of YFV into North America is also becoming a greater threat as populations of *A. aegypti* mosquitoes are repopulating areas that have ceased aggressive control efforts [15]. For similar reasons, local transmission of the *Flaviviruses* Dengue and Zika and the related alphavirus Chikungunya have already been documented in the contiguous U.S. The risk to the residents of the U.S. is particularly high with these viruses as Food and Drug Administration (FDA)-approved vaccines are currently unavailable.

The yellow fever vaccine should not simply be viewed as an empirical success that warrants no further study. 17D, arguably, sits at the pinnacle of vaccine success, alongside those for small pox and polio. The importance of 17D remains high as a reverse genetics system [16] has made the vaccine a vector for other antigens [17,18,19,20,21] and a backbone for the development of vaccines against other flaviviruses, specifically [22]. The highly efficacious and long-lasting immunity elicited by the 17D makes it an important target of future research for the development of vaccines against related viruses and the understanding attenuation and immune inductive processes for highly efficacious vaccines in general [23]. Despite the ongoing use and development of 17D, the relatively recent recognition that the vaccine causes rare but serious adverse events (SAE) has led to concerns over its continued use [24,25]. The causes of SAE are unknown, but patient-specific immune irregularities may be involved [26,27].

While the humoral immune response elicited by 17D has historically been considered protective against challenge with a virulent strain of YFV, it is unknown whether the T cell arm of the immune response plays a role. A thorough understanding of these immune responses remains the most glaring knowledge gap regarding 17D. Bridging this divide is likely to illuminate mechanisms that contribute to SAE as well as facilitate the rational design of vaccines, particularly those based off the 17D platform. The remainder of this review will briefly describe the human immune response to 17D with a focus on T cell mediated immunity. Attention will then be turned to animal models of immunity that are proving useful for characterizing the role of T cell immunity for protection against challenge with virulent YFV and the control of 17D during vaccination.

## 2. The Immune Response to Yellow Fever Virus in Humans

The abundance of vaccinated individuals and the safety of 17D has ensured ample availability of subjects for the study of human immune responses following vaccination. For example, such subjects have been derived from mass vaccination of U.S. troops during the World War II and recent targeted studies that have recruited volunteers for immunization. Subjects like the former, studied many years after vaccination, have demonstrated the striking longevity of immunity to 17D whereas the latter are opening windows into the earliest events following vaccination. These studies have been limited to evaluating what can be obtained from blood sampling. Thus, since its development, the most studied aspect of human immunity to 17D was the humoral response. However, studies of T cell-mediated immunity to 17D have become prolific in recent years.

### 2.1. Neutralizing Antibodies as Protective Immunity

Since wild-type virus cannot be used for directed experimentation on humans, empirical evaluation of specific immune components involved in protecting humans is practically non-existent. However, serum from naturally infected convalescent humans exposed to wild-type virus protects rhesus macaques against infection [4], and mice are protected against intracerebral challenge with 17D when mixed with serum from 17D-vaccinated humans [9]. Furthermore, convalescent serum collected from humans located on another continent could protect monkeys infected with local isolates of virus [28]. These studies suggested that (1) humoral immunity was a primary protective element in previously exposed individuals and (2) a single vaccine could offer protection to global strains of YFV. Thus, neutralizing antibodies remain the accepted correlate of protection against YFV.

### 2.2. The Neutralizing Antibody Response to 17D

Consistently, 90% or greater of the individuals immunized with 17D develop neutralizing antibodies (reviewed in [29]). Early studies quantified neutralizing antibody titers as dilutions of immune serum that protected 50% of intracranially infected mice against 17D [8,9]. By this method, neutralizing antibodies have been detected as early as two weeks following vaccination [9]. Plaque reduction neutralization tests (PRNT) demonstrated robust neutralizing antibody responses beginning as early as six days following vaccination [30], with most individuals peaking after thirty days [30]. Neutralizing responses have been recovered for up to 60 years after vaccination [31,32,33,34]. The longevity of the 17D-specific neutralizing antibody responses suggests that 17D elicits a long-lived and functional memory response, supported by a boost in titer after revaccination [30,35]. This and other evidence for the potency of neutralizing antibody responses has been presented in detail (reviewed in [29]), and suggests that a single vaccination offers life-long protection against YFV.

### 2.3. Innate Immune Responses to Vaccination with 17D

17D elicits a complex modulation of innate immune cytokines in humans. Elevated levels of plasma interferon (IFN)-γ are seen on day 15 post vaccination [36]. Although plasma levels of other cytokines have not been assessed, re-stimulation of innate immune cell cultures of natural killer (NK) cells, neutrophils, and monocytes from 17D vaccinated humans with YF antigen results in the increased production of IFN-γ [36], interleukin (IL)-1β [37], IL-12 [38], tumor necrosis factor (TNF)-α [38,39] and IL-10 [39] in some subsets and a concomitant decrease of TNF-α [38], IL-10, and IL-4 [39] in other subsets. A wide array of increased innate immune gene expression signatures is detected as early as one day following vaccination. The cytokines include inducers and mediators of antiviral and interferon activity such as Toll-like receptor (TLR)-7, interferon-induced protein with tetratricopeptide repeats (IFIT) 1, IFIT2, IFIT3, interferon regulatory factor (IRF) 7, and signal transducer and activator of transcription (STAT) 1 [40]. Gene signatures of directly antiviral cytokines IFN-α and IFN-γ are highly variable among vaccine recipients, with a near even distribution of vaccinees showing an increase or decrease of IFN-α and IFN-γ transcripts between days three and seven post vaccination [40].

The earliest induction of cytokines by 17D is most likely the result of direct interactions with innate immune cells like dendritic cells (DC). Stimulation of human monocyte-derived DCs and plasmacytoid DCs with the 17D vaccine preparation induces the production of IL-6, TNF-α, monocyte chemoattractant protein (MCP)-1, interferon-γ-induced protein 10 kDa (IP-10), IL-12p40, IL-12p70 and IFN-α. Murine studies suggested that the activation and production of cytokines by DCs is mediated through interactions with TLR-2, -7, -8 and -9 [41]. Engagement of multiple TLRs results in a complex relationship with the downstream T cell response, resulting in the production of both T_H_1 and T_H_2 cytokines. Although TLR-2 suppresses cytotoxic T cell responses to 17D in mice, TLR signaling more broadly promote the very same responses [41]. 17D infects and replicates only minimally in human DCs [41,42], likely due to triggering of maturation [43]. However, 17D antigens are processed and presented by DCs [43], which are most likely involved in eliciting downstream T cell responses.

### 2.4. T Cell-Mediated Immune Responses to 17D

Prior to 1998 [30] the T cell response to 17D in humans had not been studied. While a T cell response to vaccination had been expected, the role of T cells was unclear. Since neutralizing antibodies were the accepted means of protection against YFV, it was strongly expected that cluster of differentiation (CD)4+ T cells would act primarily as a form of ‘help’ during the formation of neutralizing antibody responses. CD8+ T cells recognize and lyse virus-infected cells and thus it was expected that CD8+ T cells could play a role in clearance of 17D during vaccination or for protection following challenge with wild-type YFV. The specific role that T cells play in humans during immunization, or for protection following challenge remains uncertain, and is likely to remain so due to the limitations of working with human subjects. Since the first T cell studies, a great deal of information has been gathered about the dynamics of T cell activation, proliferation, and the phenotypes that result from 17D immunization.

Both CD4+ and CD8+ T cells respond strongly to 17D. Activated cells are detected as early as three days post-immunization (CD8+) [44]. CD4+ T cells peak between 7 and 14 days after immunization [45,46,47,48], before CD8+ T cells which peak between 14 and 30 days [44,45,46,49,50]. CD8+ T cell proliferation appears to be largely antigen-driven since the size of the T cell response correlates directly with the virus load. The peak of the CD8+ T cell response comes three days after the highest levels of virus genomes are detected in the plasma, and CD8+ T cells peak only after virus is no longer detected [44]. Five to six percent of the approximately 2000 CD8+ T cell clones that respond to 17D [51] differentiate into various memory populations displaying conventional markers including central memory (T_CM_) and effector memory (T_EM_/T_EMRA_). These cells slowly decrease over time, but remain detectable for over 25 years [33,52]. In contrast, a population of self-renewing and highly responsive 17D-specific memory cells similar to the stem cell-like memory subset (T_scm_) maintains stable numbers for the same 25-year period (discussed below) [52].

#### 2.4.1. CD4+ T Cells

17D-specific CD4+ T cells produce a mix of T_H_1 and T_H_2 cytokines, including IL-2, IFN-γ, TNF-α, IL-12 (T_H_1), IL-4, IL-5, IL-10, and IL-13 (T_H_2) [39,45,47,53]. T_H_1 T cells characteristically promote CD8+ responses whereas T_H_2 cytokines promote B-cell and antibody responses. T follicular helper (Tfh) cells are a subset of CD4+ T cells characterized in part by the expression of C–X–C chemokine receptor type 5 (CXC-R5) and the production of IL-4. Tfh cells promote healthy B cell germinal centers (reviewed in [54]). It is unclear whether the CD4+ T cells provide a ‘helper’ function that promotes the production of neutralizing antibodies or CD8+ T cell responses or if these responses are independent of T cells. Whether CD8+ T cells are dependent on CD4+ T cell help, in the context of 17D immunization, has not been studied.

An antibody ‘helper’ scenario is supported by one study where a greater proportion of CD4+ IL-2 and IFN-γ producing T cells on days 1 and 2 were associated with higher neutralizing antibody titers and greater numbers of plasmablasts on day 14 [45]. Murine studies demonstrate that both T_H_1 and T_H_2 cytokines promote the generation of neutralizing antibody responses to viruses. IFN-γ favors the production of immunoglobulin G2a (IgG2a) and IL-4 favors immunoglobulin G1 (IgG1) while both cytokines promote class switching and affinity maturation (reviewed in [55]). It remains unknown whether Tfh cells play a central role in antibody development towards 17D; although in one murine model, Tfh cells may have a limited role in eliciting protective neutralizing antibodies [56].

Human CD4+ forkhead box P3 (FOXP3)+ T regulatory cells are transiently activated during immunization with 17D [46] although their relationship to the immune outcomes following 17D vaccination has not been investigated.

#### 2.4.2. CD8+ T Cells

17D-specific CD8+ T cells respond to epitopes within every mature product of the 17D polyprotein [46,50]. Upon peptide re-stimulation, 17D-specific CD8+ T cells produce multiple cytokines including proinflammatory cytokines IFN-γ, TNF-α, macrophage inflammatory protein (MIP1)-β [46,50] and the proliferation promoting cytokine IL-2 [46]. Nearly all 17D-specific CD8+ T cells express granzyme B, and the majority of stimulated cells stain positive for surface CD107a [50], suggesting that they are functionally capable of degranulating [57] and are likely to be cytolytic. Following the peak of CD8+ T cell expansion, the cells begin to differentiate in to long-lived memory which retain a polyfunctional phenotype for at least two years [50].

Memory CD8+ T cells are composed of conventional T_CM_ (CCR7+, CD45RA−), T_EM_ (CCR7−, CD45RA−) and T_EMRA_ (CCR7−, CD45RA+), all of which are induced strongly by vaccination and slowly decrease over a 25+ year period. However, a recently identified population of the ‘naïve-like’ T_scm_ was shown to persist at nearly unchanged levels for the same 25-year period. These cells appear naïve according to CCR7+, CD45RA+ expression but, unlike truly naïve cells, they also express CD58 and CD95. The 17D-specific T_scm_ cells demonstrate a self-renewing behavior and rapid proliferation in response to re-stimulation [52]. These data suggest that 17D-specific memory T cells can respond quite rapidly to re-exposure of YFV antigen in vitro; however, it is unclear whether this happens in humans. One study demonstrated that biomarkers of T cell activity, neopterin and β2-microglobulin, do not increase after a 17D boost, whereas a strong increase is seen during the primary vaccination [33]. Another study indicated that, compared to individuals that had only one vaccination, multiple vaccinations do not result in higher levels of memory T cells as is typical following a secondary memory T cell response [30].

### 2.5. Naïve Host Infection with Wild-Type YFV: A Limited Understanding of Human Immunity

Very few studies have evaluated the immune response to a primary infection with virulent YFV. Most infections are only identified when patients are in fulminant hemorrhagic disease, if not after death. Thus, much of the information regarding immunity towards wild-type infection is from severe cases of disease and not from the majority, for which the immune response clears the infection. In one study, 18 patients with non-fatal non-hemorrhagic YF were more likely to have measurable neutralizing antibody titers and undetectable virus by reverse transcription polymerase chain reaction (RT-PCR) compared to fatal or non-fatal hemorrhagic cases. Cases of severe disease have elevated chemoattractant cytokines MCP-1 and IP-10 and proinflammatory cytokines TNF-a, IL-6, and IL-1a [58]. These data suggest that size of the neutralizing antibody response to wild-type YFV infection may be a factor determining the severity of disease. Therefore, elevated levels of cytokines seen in severe cases of disease may be the result of virus burden in individuals that mount an insufficient neutralizing antibody response.

Livers from patients succumbing to fatal yellow fever show apoptosis, steatosis and lytic necrosis of hepatocytes. This pattern is associated with a strong infiltration of T cells, primarily CD4+, but also CD8+ T cells [59,60] and B cells into the portal region [59]. Macrophages, NK cells and antigen presenting cells also infiltrate the liver, but in fewer numbers [60]. Expression of TNF-α, IFN-γ, and transforming growth factor (TGF)-β were elevated most strongly in the lobular regions that showed strong macrophage infiltration [59]. The heavy T cell infiltration associated with cell death and elevated cytokine expression suggests that severe disease may have a prominent immunopathologic component [60,61]. These data suggest that patients presenting with severe disease mount a T cell response that may be either (1) ineffective at controlling virus replication in the liver or (2) is strongly cytolytic, causing damage and exacerbating disease.

## 3. Animals Models of T Cell-Mediated Immunity towards YFV

Human studies are ideal for recording the phenotypes and reactivity of developing or developed immunity to 17D. Retrospective studies have provided an insight into the statistical effectiveness of vaccination and the resulting immune phenotypes. However, questions about mechanisms of protection and anti-YFV immune development cannot currently be asked in humans. Human volunteers can neither be experimentally infected with wild-type YFV nor have their visceral or neurological tissues probed for the presence of virus and immune mediators. Thus, a more thorough understanding of the virus–host interactions must be addressed using animal models.

### 3.1. Non-Human Primates

Non-human primates serve as the most relevant models of YF due to their close relationship to humans and their natural susceptibility to infection. Rhesus macaques develop severe hemorrhagic yellow fever, presenting with symptoms that resemble human disease [4]. Vaccination with 17D induces neutralizing antibodies that correlate with protection against a virulent YFV [62]. However, only a few studies have addressed T cell responses to 17D in these animals. Activated T cells peak between 12 and 14 days following immunization [63]. CD8+ T cells respond to peptides throughout the genome of 17D. Low levels of CD4+ T cells can be detected as well [64]. 17D-specific T cells produce IFN-γ, IL-4 and TNF-α in response to peptide stimulation. A majority of IFN-γ producing CD8+ T cells may be γδ T cell receptor (TCR)+ [65].

### 3.2. Murine Models

Murine models are particularly advantageous for studies of cellular immunity due to known major histocompatibility complex (MHC) haplotypes, well characterized biology and the ubiquitous availability of reagents. However, few studies have addressed the development or role of T cell-mediated responses to YFV in murine model systems. This is primarily because YFV is refractory to replication in adult mice due to restriction by type-I interferon [66] (with the exception of the central nervous system when introduced by intracranial inoculation [6]). Thus, when virus is introduced into the mouse through a peripheral route that mimics vaccination—e.g., subcutaneous (SC)—, little to no replication is detected [67]. Adaptive immune responses play no known role in eliminating virus from mice infected via this route, as infection of recombination activation gene (RAG)^−/−^ animals lacking functional B cells and T cells does not result in the development of disease [56]. Mice do mount an immune response to 17D when it is administered SC that includes neutralizing antibodies and CD8+ and CD4+ T cells [56,68,69]. However, 17D-specific T cells proliferate modestly in this model, compared to humans where responses to specific epitopes can account for 10–20% of total CD8+ T cells [46,49], most likely due to the lack of virus replication which limits viral antigen. In 17D-immunized mice, neutralizing antibodies and CD8+ T cells protect mice against intracranial challenge with 17D through an IFN-γ and perforin mediated mechanism [56]. A similar mechanism is observed when 17D-derived antigens are delivered by an unrelated viral vector [70]. These studies suggest that CD8+ T cells and neutralizing antibodies may be important for the clearance of 17D from the central nervous system (CNS) during vaccination. No studies have been published that examine clearance of the wild-type virus in this model. Although, wild-type virus and 17D are equally virulent by intracranial (IC) infection [71], making it difficult to differentiate the two strains experimentally. The virulence of 17D following IC infection suggests that mice may be a useful model of yellow fever vaccine-associated neurotropic disease (YEL-AND), a rare SAE seen in human vaccinees. These studies suggest that an inadequate neutralizing antibody or CD8+ T cell immune response may increase susceptibility to YEL-AND.

YFV replication in mice is primarily restricted by type-I interferon and to a lesser extent type-II interferon [66]. In type-I interferon receptor knockout mice (IFNAR^−/−^), which lack all type-I interferon responses, YFV replicates when introduced by the SC route. Notably, 17D and wild-type viruses can be differentiated by both replication and clinical disease in the IFNAR^−/−^ mouse. 17D remains attenuated with replication restricted to the lymphoid compartments including the lymph nodes, spleen, bone marrow and blood, whereas the wild-type virus produces a viscerotropic disease resembling that seen in humans, including virus replication, immune infiltrates and steatosis in the liver. Like 17D, wild-type virus also replicates in lymphoid tissues but additionally in the kidneys, heart, and brain [66,67]. Importantly, 17D is cleared from all IFNAR^−/−^ animals whereas wild-type virus is lethal. Furthermore, 17D immunization protects mice against challenge with a wild-type virus. The mechanisms of 17D clearance in IFNAR^−/−^ mice is not known, but unpublished observations [72] indicate that CD8+ T cells are required, consistent with studies of IC inoculation in normal mice.

The IFNAR^−/−^ model is not ideal for the study of T cell responses. Depending on the pathogen, type-I interferon can significantly influence the activation and proliferation of T cells [73,74]. Type-I interferon is produced by human and rhesus macaque cells infected with 17D in vitro [41,42,75,76,77,78]; however, a role for type-I interferon in YFV-specific adaptive immune responses in vivo has not been defined. Despite these concerns, 17D-specific T cells induced in IFNAR^−/−^ mice appear to be normal, and their phenotypes align well with those seen in human studies. The T cells are polyfunctional, producing combinations of IFN-γ, TNF-α (CD8+, CD4+) and IL-2 (CD8+) and both CD4+ and CD8+ T cells present CD107a on their surface during peptide stimulation and functionally lyse 17D-specific targets in vivo. The T cells contract into long-lived memory that persists for at least two and a half years, essentially for the life of the mouse. In this model, adoptive transfer experiments determined that both neutralizing antibodies and CD4+ T cells contribute to protection. Surprisingly, no protective effect was seen with CD8+ T cells [67]. This study, when considered with the IC model of YEL-AND, suggests that CD8+ and CD4+ T cells may be differentially important in regard to protection of naïve animals against YEL-AND (CD8+ T cell-mediated), and protection following challenge of vaccinated animals with a virulent strain of YFV (CD4+ T cell-mediated).

When both type-I and type-II interferon receptors are absent (IFNAGR^−/−^), mice become susceptible to disease from both 17D [66,79] and the wild-type virus [66]. Thus, 17D is attenuated by IFN-γ. However, the disease associated with these viruses is dramatically different. Wild-type YFV infected IFNAGR^−/−^ mice develop an accelerated viscerotropic disease, like that seen in IFNAR^−/−^ mice, with an average survival time that is one day less (6–7 days post infection) than in the presence of type-II interferon signaling. 17D infected IFNAGR^−/−^ mice develop a protracted infection that resembles a mild viscerotropic-like disease at the onset but mice then succumb to a disease associated with neurological signs 12 days post infection [66]. The development of neurologic disease suggests that IFNAGR^−/−^ mice may be a useful model of YEL-AND. Although adaptive immune responses to 17D in IFNAGR^−/−^ mice have not been studied, one possible mechanism resulting in neurologic disease is the ineffective clearance of 17D from the brain since infected cells in the brain cannot respond to IFN-γ. This would be consistent with the role that IFN-γ producing CD8+ T cells play in clearance of 17D from IC infection of normal mice [56].

The IFNAGR^−/−^ model can be partially reproduced using a conditional knockout of STAT1, a primary signaling adapter of type I and type II interferon responses [80,81]. This model (Stat1^loxP/loxP^/Vav-cre) [82] can be thought of as a chimeric model where visceral organs respond competently to type I and type II interferons but the response in cells of the hematopoietic lineage is blunted. In this model, 17D replicated in every hematopoietic cell type tested in the blood and spleen, indicating that 17D is tropic for these cell types in mice, absent interferon responses. Unlike human vaccination, 17D was introduced intravenously and proved lethal to approximately 75% of mice over a time-frame similar to that seen in IFNAGR^−/−^ mice. Virus genomes were identified in the liver, kidney and brain at the same levels as those seen in wild-type mice. Consistent with STAT1 deficiency among hematopoietic cells, the spleen showed significantly higher levels of 17D genomes than the other organs. These data suggest that lymphocytic infiltrates and inflammation that was identified in the liver, may be due to non-specific immunopathologic mechanisms, perhaps cytokine-induced. Signs of viscerotropic disease (e.g. liver pathology) may make this a useful model for understanding the contribution of immune pathology to YFV-AVD. In this model, both CD4+ and CD8+ T cells proliferated in response to infection, with CD4+ T cells becoming most enriched, and both cell types displaying markers of memory differentiation. Among the minority of animals that survived infection, it is unclear if T cells contributed to survival or what role T cells played in the clearance of virus. Although, virus was mostly cleared from the peripheral blood at day eleven post-infection (the latest timepoint studied), replication in the spleen continued. An analogous model where the murine hematopoietic system was reconstituted with (type I and type II interferon competent) human cells, 17D replicated in all human cell types tested and caused minimal non-lethal disease. T cells became activated with minor proliferation, but their role in the clearance of virus was not assessed.

## 4. Conclusions

The 17D-induced adaptive immune response in humans has been well characterized in recent years. There is a robust induction of both neutralizing antibodies and T cells, and both responses have been detected up to three decades following a single immunization. Neutralizing antibodies are the primary correlate of protections in vaccines and animal studies suggest that neutralizing antibodies can be sufficient for protection against challenge. Yet it remains unclear whether T cells also serve a protective function following challenge. Human 17D-specific CD4+ and CD8+ T cell compartments are polyfunctional, suggesting that they may play a role in protection following challenge. However, no studies have addressed this question in humans, and it is unlikely that it can ever be addressed without the use of experimental animal models.

Non-human primates (NHP) are by far the best experimental models for of YFV due to being a natural host and closely related to humans. NHP models have demonstrated the importance of neutralizing antibodies for protection following challenge with virulent YFV. T cells become activated and expand in response to vaccination; although, it is unclear whether they are functionally protective. Only a few studies have addressed the T cell response in these animals. This is not surprising since the outbred nature of NHPs—resulting in variety of MHC haplotypes—makes the study of specific T cell responses difficult. Since two individual NHPs are unlikely to be MHC compatible, complex experiments like adoptive transfers are not feasible. This shortcoming could be addressed with innovative strategies like partial MHC haplotype adoptive transfer approaches [83], the use of novel models like marmosets which give birth to MHC chimeric twins [84] or future mastery of genetic engineering in NHPs.

With regards to T cell immune studies, murine models hold the most promise due to the availability of highly characterized inbred animals and available reagents. IFNAR^−/−^ mice differentiate between 17D and wild-type virus regarding the extent of attenuation and disease. This model has been used to demonstrate that the immunity elicited by 17D is proportional to that found in humans regarding magnitude, function, and long-lived memory. The flexibility of the inbred mouse enables complex immune studies, and therefore this is the only model where 17D-specific T cells have been shown to exhibit protective efficacy during challenge with a wild-type virus. The IC infection model of YEL-AND has demonstrated the importance of CD8+ T cells for clearance of virus from the brain. An alternative model of YEL-AND is the IFNAGR^−/−^ mouse in which 17D can be delivered SC, to simulate human vaccination. However, ultimately, murine immunodeficient models are not without substantial concern. In normal mice, IC inoculation of 17D is not a physiological route of exposure but is required to induce disease. Additionally, the lack of proliferation when 17D is administered by routes other than IC is not consistent with human vaccination and may result in T cell phenotypes that do not fully resemble human T cells. The IFNAR^−/−^ model demonstrates YFV replication outside of the CNS, but may have deficiencies in the magnitude and the quality of the adaptive immune response.

17D continues to be administered to travelers and in YFV endemic countries as the only prevention for YF. 17D is also being used as a vector for the delivery of foreign antigens against the related dengue virus, and this approach has been licensed in Mexico. 17D continues to be developed for the delivery of novel antigens aimed at protection against other pathogens like human immunodeficiency virus (HIV), malaria, trypanosomes and the Lassa virus [17,18,19,20,21]. The risk of SAE in these chimeric 17D viruses remains unknown. Moreover, the mechanisms by which the 17D vaccine is cleared and mediates protection against a virulent virus is not well understood. Establishing an understanding of these mechanisms serves both to prevent cases of SAE and improve upon 17D-chimeric vaccines. 17D has many secrets yet to be revealed regarding factors that contribute to vaccine efficacy, and most of these questions cannot be asked using human studies. Animal models provide the only means to assess the primary immune response to virulent YFV, the protective immunity elicited by 17D, map mutations that contribute to the attenuation and immunogenicity of 17D, and, perhaps most importantly, facilitate the improvement of chimeric vaccines regarding both safety and immunogenicity towards delivered antigens. The authors acknowledge that existing animal models for YFV are less than ideal; however, for the sake of progress, it remains important that the scientific community embrace these types of models and continually work towards their improvement.
